# Physical activity and 4-year changes in body weight in 52,498 non-obese people: the Lifelines cohort

**DOI:** 10.1186/s12966-021-01141-8

**Published:** 2021-06-07

**Authors:** Oyuntugs Byambasukh, Petra Vinke, Daan Kromhout, Gerjan Navis, Eva Corpeleijn

**Affiliations:** 1 Department of Epidemiology (FA40), Unit of Lifestyle Medicine in Obesity and Diabetes, University Medical Center Groningen, University of Groningen, P.O. Box 30 001, 9700 RB Groningen, the Netherlands; 2 Department of Endocrinology, Mongolian National University of Medical Sciences, Ulaanbaatar, Mongolia; 3 Department of Internal Medicine, University Medical Center Groningen, University of Groningen, Groningen, the Netherlands

**Keywords:** Physical activity, Weight gain prevention, Moderate-to-vigorous physical activity, Leisure-time physical activity, Occupational physical activity, Life course

## Abstract

**Objectives:**

We investigated associations between leisure-time physical activity (LTPA) at different intensities (moderate and vigorous or moderate-to-vigorous) and prospective weight gain in non-obese people. We also examined whether these associations were independent of other lifestyle factors and changes in muscle mass and whether they were age-dependent and changed over a person’s life course.

**Methods:**

The data were extracted from the Lifelines cohort study (*N* = 52,498; 43.5% men) and excluded obese individuals (BMI > 30 kg/m^2^). We used the validated SQUASH questionnaire to estimate moderate-to-vigorous (MVPA; MET≥4), moderate (MPA; MET between 4 and 6.5) and vigorous PA (VPA; MET≥6.5). Body weight was objectively measured, and changes were standardized to a 4-year period. Separate analyses, adjusted for age, educational level, diet, smoking, alcohol consumption and changes in creatinine excretion (a marker of muscle mass), were performed for men and women.

**Results:**

The average weight gain was + 0.45 ± 0.03 kg in women. Relative to each reference groups (No-MVPA, No-MPA and No-VPA), MVPA (Beta (95%CI): − 0.34 kg (− 0.56;-0.13)), MPA (− 0.32 kg (− 0.54;-0.10)) and VPA (− 0.30 kg (− 0.43;-0.18)) were associated with less gain in body weight in women after adjusting for potential confounders, described above. These associations were dose-dependent when physically active individuals were divided in tertiles. Beta-coefficients (95%CI) for the lowest, middle, and highest MVPA tertiles relative to the ‘No-MVPA’ were, respectively, − 0.24 (− 0.47;-0.02), − 0.31 (− 0.53;-0.08), and − 0.38 (− 0.61;-0.16) kg. The average weight gain in men was + 0.13 ± 0.03 kg, and only VPA, not MPA was associated with less body weight gain. Beta-coefficients (95%CI) for the VPA tertiles relative to the ‘No-VPA’ group were, respectively, − 0.25 (− 0.42;-0.09), − 0.19 (− 0.38;-0.01) and − 0.20 (− 0.38;-0.02) kg. However, after adjusting for potential confounders, the association was no longer significant in men. The potential benefits of leisure-time PA were age-stratified and mainly observed in younger adults (men < 35 years) or stronger with younger age (women < 55 years).

**Conclusion:**

Higher leisure-time MVPA, MPA, and VPA were associated with less weight gain in women < 55 years. In younger men (< 35 years), only VPA was associated with less weight gain.

**Supplementary Information:**

The online version contains supplementary material available at 10.1186/s12966-021-01141-8.

## Introduction

Obesity contributes to the development of a number of chronic diseases, such as type 2 diabetes, cardiovascular diseases, and certain cancers [1]. Obesity rates among adults nearly trebled between 1975 and 2016 [2], and the epidemic proportions of obesity and obesity-related diseases continue to pose major health problems, globally. The global rate of type 2 diabetes among adults rose from 4.7% in 1980 to 8.5% in 2014 [1]. while one-third of all deaths worldwide are attributed to cardiovascular diseases [3]. With obesity acknowledged as the underlying cause of these health concerns, attention has shifted to the primordial prevention of obesity in non-obese people, necessitating the development and improvement of strategies for preventing weight gain [4, 5]. Genetic, socio-economic, and environmental factors generally account for body weight gain [6–8]. These factors influence energy balance-related behaviours that determine energy intake and expenditure. The primordial prevention of excessive calorie intake and of low levels of energy expenditure (i.e., low physical activity) constitute the main strategy for reducing the risk of weight gain [7, 9].

Previous studies have mainly focused on the benefits of increased physical activity (PA) as a strategy for promoting body weight loss and for preventing the regaining of body weight in obese individuals [10]. They have shown that individuals who become more active lose more body weight. Several large-scale studies have found that PA plays a role in the prevention of body weight gain [11–13]. By contrast, other studies have found no association between baseline PA and changes in body weight during follow-up assessments [14–17]. In some studies that mostly included small sample sizes, this association was only observed in subgroups, for example, in normal weight, female, or younger adults [18, 19]. Therefore, large-scale population-based studies that test the benefits of PA across groups differentiated by age and sex are required. Moreover, little is known about how the intensity and type of daily-life PA impact on its association with prospective changes in body weight. Clinical guidelines on physical activity recommended that people should do at least 150 min of moderate-intensity, or 75 min of vigorous intensity PA a week [20]. Although clinical guidelines recommend that physical activities should be conducted at moderate-to-vigorous and not at light intensity levels, most previous studies focused on total PA, including light PA [14, 15, 18, 19, 21]. Moreover, there are still unanswered questions as to whether vigorous PA is necessary for achieving a health benefit. As reported in the literature, VPA has more impact on VO^2^_max_ and fitness level and induces more release of growth hormone and catecholamines, suggesting that benefits of physical activity differ according to intensity level, such that vigorous PA may affect muscle mass more, and also increase basal metabolic rate more, than moderate and light PA [22, 23]. However, not all individuals are able or willing to perform vigorous PA, and this may not even be necessary if a moderate intensity level is in fact effective.

The primary objective of our study is to determine associations between leisure-time physical activity (LTPA) at different intensities (moderate and vigorous or moderate-to-vigorous) and prospective weight gain in non-obese people. We also examined whether these associations are independent of other lifestyle factors and changes in muscle mass and whether they are age-dependent and change over a person’s life course. In case that the association of MVPA depends on its intensity, we explore how it relates to the types of activities reported by the participants for translational purposes.

## Methods

### Data source and study population

Lifelines is a multidisciplinary prospective population-based cohort and biobank of more than 167,000 people living in the North of the Netherlands [21]. It employs a broad range of investigative procedures in assessing the biomedical, socio-demographic, behavioral, physical, and psychological factors that contribute to the health and disease of the general population, with a special focus on multi-morbidity and complex genetics. The study is conducted according to the Helsinki Declaration, and it was approved by the medical ethical committee of the University Medical Center Groningen in the Netherlands. All participants provided their written informed consent [24].

**Fig. 1:**
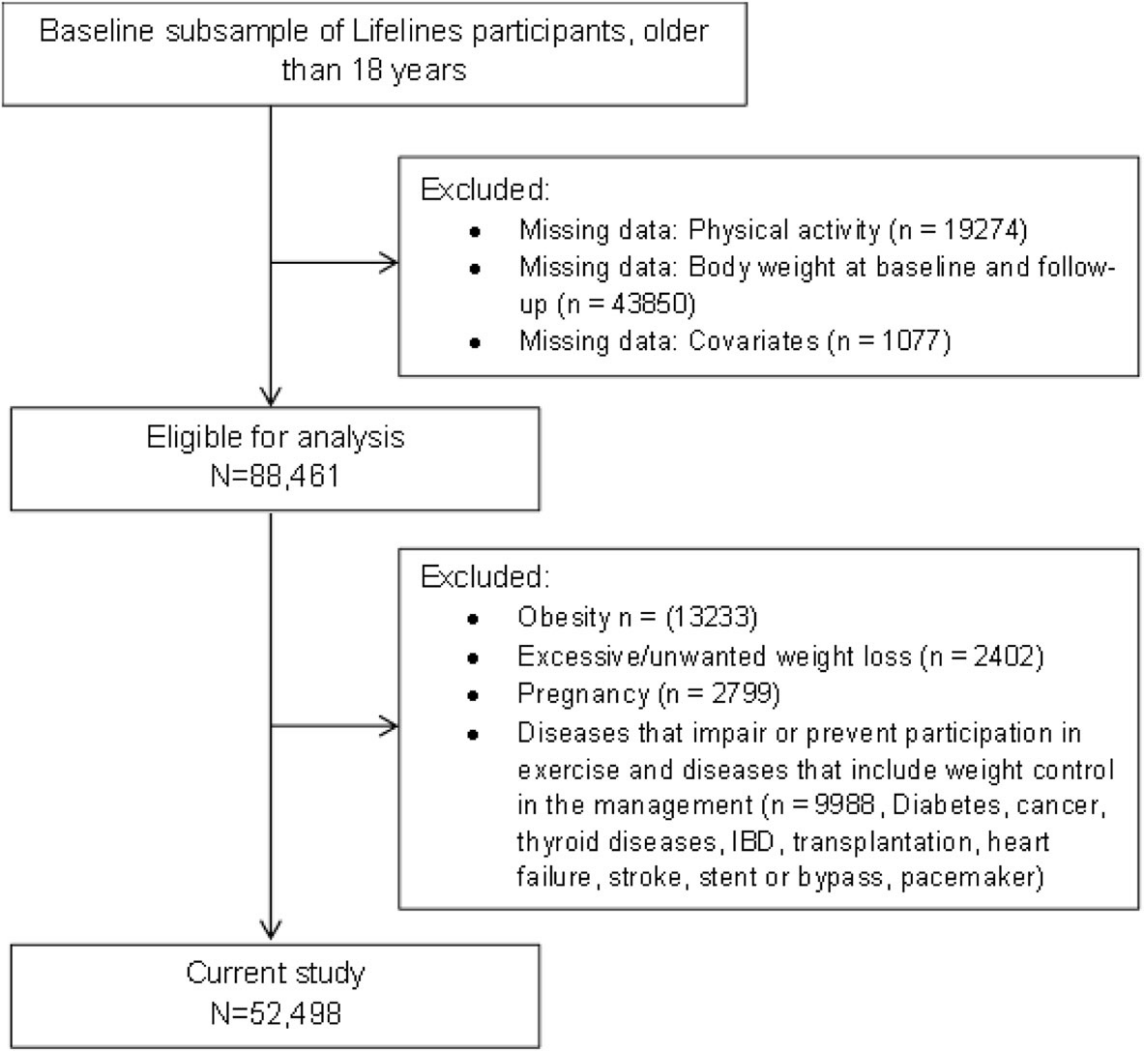
Flowchart of the study population

In this study, the analyses were based on the data at baseline and at 4-year follow-up. We included non-obese (BMI < 30 kg/m^2^) adult (> 18 years) subjects of Western European origin. The first exclusion was any missing and/or implausible data related to the main determinant and outcome: assessment of physical activity and the measurements of body weight. Further exclusions were related to minimize bias from changes in physical activity or body weight: excessive or unwanted weight loss, pregnancy, type 2 diabetes, thyroid diseases, irritable bowel syndrome, transplantation, cancer, heart failure, stroke, stent or bypass and pacemaker. In all, 52,498 participants were included in the current analyses (Fig. 1).

### Assessment of physical activity

Physical activity was assessed using the Short QUestionnaire to ASsess Health-enhancing (SQUASH) physical activity, a validated questionnaire, which estimates habitual physical activities with reference to a normal week in the past month [25]. The SQUASH questionnaire allows for the categorization of minutes of PA according to levels of intensity, namely light, moderate, and vigorous. Furthermore, another reason for having chosen this questionnaire in this study is that the SQUASH allows to measure PA minutes through intensity level and through different types of daily-life PA at the same time. The SQUASH is pre-structured into four domains: commuting, leisure time, household, and occupational activities. Questions consisted of three main queries: days per week, average time per day, and intensity. Each activity in minutes per week was calculated by multiplying frequency (days/week) by duration (min/day). Then, the activities were assigned to a certain level of effort, or intensity, indicated by the MET value of the activity [25–27] MET values were assigned to activities with the help of Ainsworth’s Compendium of Physical Activities [28].

In this study, leisure-time (including commuting) moderate-to-vigorous PA (MVPA; MET ≥4.0), moderate PA (MPA; MET between 4 and 6.5) and vigorous PA (VPA; MET ≥6.5) categories were used as the main measures of physical activity. These intervals are applied according to the guidelines for Dutch physical activity [29]. Participants were divided into distinct categories based on the amount of MVPA, MPA and VPA. Individuals who performed no physical activity at MVPA, MPA and VPA, respectively were classified as ‘No-MVPA’, ‘No-MPA’ and ‘No-VPA’ (T0). The other participants (MVPA> 0 min/week, MPA > 0 min/week and VPA > 0 min/week) were divided into distinct tertiles of MVPA, MPA and VPA ranging from low (tertile 1, T1), middle (tertile 2, T2) to high (tertile 3, T3). Thus, T0, T1, T2 and T3 were considered as ‘inactive’, ‘a little bit active’, ‘active’, and ‘very active’ respectively [26].

Additionally, activity minutes per week for specific types of daily-life physical activity (walking, cycling, sports and odd jobs) at moderate or vigorous intensity were categorized into two levels: No-MPA and MPA > 0 min per week, or No-VPA and VPA > 0 min per week.

### Body weight measurement

Participants’ body weights (in kg) were measured by well-trained assistants who are permanent staff members using a standardized protocol [24]. At a follow-up session conducted after 4 years, their body weights were measured to the nearest 0.1 kg using the same baseline protocol. Changes between baseline and follow-up measurements were standardized to a 4-year period.

### Other baseline measurements

Body height, waist circumference and blood pressure were measured by trained assistants at baseline, and BMI (kg/m^2^) was calculated. Blood samples were collected in the fasting state and analyzed on the day of collection at the Department of Laboratory Medicine of the University Medical Center Groningen, the Netherlands (Supplementary method 1, Additional file 2) [24].

The supplementary methods section (Supplementary method 2,  Additional file 2) provides definitions for the covariates. In brief, education levels were categorized as low, medium, and high. Current smoking status was categorized as non-smokers and smokers. Daily caloric and alcohol intakes were calculated using the Food Frequency Questionnaire and presented as kilocalories per day (kcal/day) and grammes of alcohol per day (g/day). Diet quality was assessed using the Lifelines Diet Score, which is described in greater detail elsewhere [30]. Creatinine excretion was calculated as the mean value derived from two urine samples collected over a 24-h period [31]. The method applied for analysing the urine samples is described in detail elsewhere [24].

### Statistical analysis

The study characteristics were expressed as means with a standard deviation for normally distributed variables or as medians with interquartile range (25th to 75th percentile) for non-normally distributed variables and numbers with percentages in case of categorical data. The differences between groups were compared using 1-way analysis of variance tests or Kruskal-Wallis tests for continuous variables. The frequency distributions of categorical variables were analyzed using the Pearson Chi-Square test. Furthermore, estimated changes in body weight were estimated according to the level of physical activity (MVPA and VPA) using age and education adjusted ANOVA. Outcomes were presented as mean of kilogram body weight with standard error.

Linear regression analysis was performed to evaluate the association between PA and changes in body weight. First, we investigated MVPA, MPA and VPA dichotomously (No-PA and PA > 0). In the main analysis, dummy exposure variables were created to compare each tertile of MVPA, MPA and VPA (T1–3) with the reference group (No-MVPA, No-MPA and No-VPA). Outcomes were presented as unstandardized beta-coefficients with 95% confidence intervals (95%CI). In the regression analyses, the basic model was adjusted for age and education level. In model 1, we added diet (LLDS for diet quality and daily caloric intake for diet quantity), current smoking (yes/no) and alcohol consumption (g/day) as potential lifestyle confounders to the basic model. Model 2 was adjusted for changes in creatinine excretion, a marker of muscle mass, in addition to adjustments in model 1. Model 3 included an additional sensitivity analysis on non-leisure time PA (occupational MVPA). All the regression analyses were repeated stratified by age categories (18–34, 35–54, and **≥** 55 years). Furthermore, the role of specific types of daily-life physical activities was investigated by repeating the analyses with the physical activity determinant divided into its underlying components (e.g. walking, cycling, sports etc.).

All statistical analyses were performed using IBM SPSS V.22.0 (Chicago, IL) and GraphPad Prism V.4.03 (San Diego, CA). A two-sided statistical significance was set at *p* < 0.05 for all tests.

## Results

In this study, we included 52,498 non-obese participantsof the Lifelines cohort study. Female participants were more likely to have healthy lifestyles at baseline. Fewer women were smokers or consumed alcohol, and their diet scores were healthier than those of men (Table 1). Of the participants, 13.3% of males (*n* = 3035) and 8.8% of females (*n* = 2519) did not perform any activities at a moderate-to-vigorous level (No-MVPA). Inactive participants had more often a lower educational level, lower diet score, higher cholesterol and were more often smokers. Men’s PA levels (min/week), and especially VPA were significantly higher than those of women. Table S1 shows the participants’ characteristics stratified by age. The age- and education-adjusted leisure-time MVPA mean values for men and women in min/week were, respectively, 288.0 ± 1.9 and 279.4 ± 1.7, and those for leisure-time VPA were 133.4 ± 4.2 and 88.8 ± 1.1, respectively. Figure S1 shows the levels of specific types of leisure-time MVPA.

**Table 1:** General characteristics of the study population by leisure-time MVPA level in males and females

Characteristics	Total	According to MVPA level			
		T0	T1	T2	T3
**Men**					
MVPA min/week	288.0 ± 1.9^a^	0	3–160	162–360	361–4015
Number (%)	22,827 (43.5)	3035 (13.3)	6597 (28.9)	6888 (30.2)	6307 (27.6)
Age (years)	45 (36–52)	45 (37–51)	44 (37–51)	45 (36–51)	46 (35–56)
Education:					
Lower (%, n)	24.7 (5630)	36.4 (1195)	24.2 (1597)	21.3 (1465)	23.2 (1463)
Middle (%, n)	38.5 (8784)	42.0 (1275)	40.4 (2668)	36.7 (2525)	36.7 (2316)
Higher (%, n)	36.9 (8413)	21.6 (655)	35.3 (2332)	42.1 (2898)	40.1 (2528)
Current smoking, (%, n)	20.6 (4695)	30.3 (1192)	21.5 (1349)	17.8 (1112)	16.4 (1057)
Alcohol use, (gr/day)	6.9 (2.7–15.5)	6.6 (2.2–15.8)	6.8 (2.7–14.9)	6.9 (3.1–15.6)	7.6 (2.8–15.8)
Lifelines Diet score	22.7 ± 5.63	21.3 ± 5.56	22.5 ± 5.48	23.3 ± 5.59	23.5 ± 5.67
Energy intake (kcal/day)	2406.7 ± 621.1	2426.7 ± 641.0	2405.9 ± 610.4	2363.7 ± 595.6	2443.1 ± 644.9
Body weight (kg)	84.7 ± 9.95	85.1 ± 10.3	85.0 ± 9.93	84.8 ± 9.85	84.2 ± 9.89
BMI (kg/m^2^)	25.2 ± 2.5	25.6 ± 2.5	25.3 ± 2.5	25.2 ± 2.4	25.1 ± 2.4
Waist circumference (cm)	92.2 ± 8.2	94.0 ± 8.2	92.9 ± 8.1	92.0 ± 8.0	90.7 ± 8.1
Systolic BP (mmHg)	129.0 ± 13.2	130.1 ± 13.3	129.2 ± 13.2	128.6 ± 12.9	128.5 ± 13.3
Diastolic BP (mm Hg)	76.0 ± 9.1	77.1 ± 9.0	76.3 ± 9.1	75.9 ± 9.0	75.5 ± 9.2
Total cholesterol (mmol/L)	5.18 ± 0.98	5.27 ± 0.99	5.20 ± 0.98	5.15 ± 0.96	5.14 ± 0.98
HDL-cholesterol	1.35 ± 0.31	1.28 ± 0.31	1.32 ± 0.30	1.36 ± 0.31	1.41 ± 0.33
Triglycerides (mmol/L)	1.09 (0.8–1.5)	1.21 (0.86–1.8)	1.14 (0.82–1.6)	1.08 (0.8–1.5)	1.02 (0.8–1.4)
Plasma glucose (mmol/L)	5.01 ± 0.46	5.08 ± 0.46	5.03 ± 0.46	5.01 ± 0.46	4.98 ± 0.45
**Women**					
MVPA min/week	279.4 ± 1.7^a^	0	3–150	151–330	331–3440
Number (%)	29,671 (56.5)	2599 (8.8)	9200 (31.0)	8979 (30.3)	8893 (30.0)
Age (years)	45 (38–52)	45 (38–50)	44 (37–50)	45 (38–52)	47 (39–57)
Education:					
Lower (%, n)	26.7 (7924)	33.3 (865)	25.1 (2308)	24.5 (2203)	28.7 (2548)
Middle (%, n)	46.1 (12,333)	41.4 (1076)	42.6 (3918)	41.3 (3711)	40.8 (3628)
Higher (%, n)	31.7 (9414)	25.3 (658)	32.3 (2974)	34.1 (3065)	30.6 (2717)
Current smoking, (%, n)	17.5 (5206)	29.8 (1132)	19.0 (1672)	15.0 (1410)	13.0 (1008)
Alcohol use, (gr/day)	2.9 (0.7–7.2)	2.5 (0.3–7.1)	2.7 (0.6–6.9)	3.1 (0.8–7.2)	3.4 (0.8–8.8)
Lifelines Diet score	25.4 ± 6.00	23.6 ± 6.07	24.7 ± 5.84	25.7 ± 5.83	26.5 ± 6.11
Energy intake (kcal/day)	1861.6 ± 456.2	1796.7 ± 461.0	1873.1 ± 451.1	1863.4 ± 443.9	1867.0 ± 470.0
Body weight (kg)	69.8 ± 8.96	70.1 ± 9.30	70.2 ± 9.14	69.7 ± 8.85	69.3 ± 8.69
BMI (kg/m^2^)	24.2 ± 2.8	24.5 ± 2.8	24.3 ± 2.8	24.2 ± 2.7	24.0 ± 2.7
Waist circumference (cm)	83.1 ± 8.8	84.5 ± 9.0	83.7 ± 8.9	83.0 ± 8.8	82.2 ± 8.6
Systolic BP (mmHg)	120.7 ± 14.6	122.3 ± 14.9	120.2 ± 14.2	120.5 ± 14.7	120.9 ± 14.8
Diastolic BP (mm Hg)	71.4 ± 8.6	72.3 ± 9.0	71.2 ± 8.5	71.4 ± 8.6	71.3 ± 8.7
Total cholesterol (mmol/L)	5.08 ± 0.99	5.09 ± 0.97	5.01 ± 0.98	5.08 ± 0.98	5.16 ± 1.03
HDL-cholesterol	1.68 ± 0.39	1.62 ± 0.39	1.64 ± 0.38	1.69 ± 0.38	1.74 ± 0.41
Triglycerides (mmol/L)	0.83 (0.6–1.1)	0.89 (0.67–1.2)	0.84 (0.63–1.1)	0.82 (0.6–1.1)	0.81 (0.6–1.1)
Plasma glucose (mmol/L)	4.76 ± 0.44	4.77 ± 0.43	4.75 ± 0.43	4.76 ± 0.44	4.76 ± 0.45

Data are presented as mean ± SD or median (25th to 75th percentile) and number (percentages, %). *Abbreviations*: *BMI* body mass index, *BP* blood pressure, *HDL-C* high-density lipoprotein cholesterol, *HbA1c* hemoglobin-A1c, *MVPA* moderate-to-vigorous physical activity, *VPA* vigorous physical activity, *T* tertile

^a^age- and education- adjusted mean ± standardized error

**Fig. 2:**
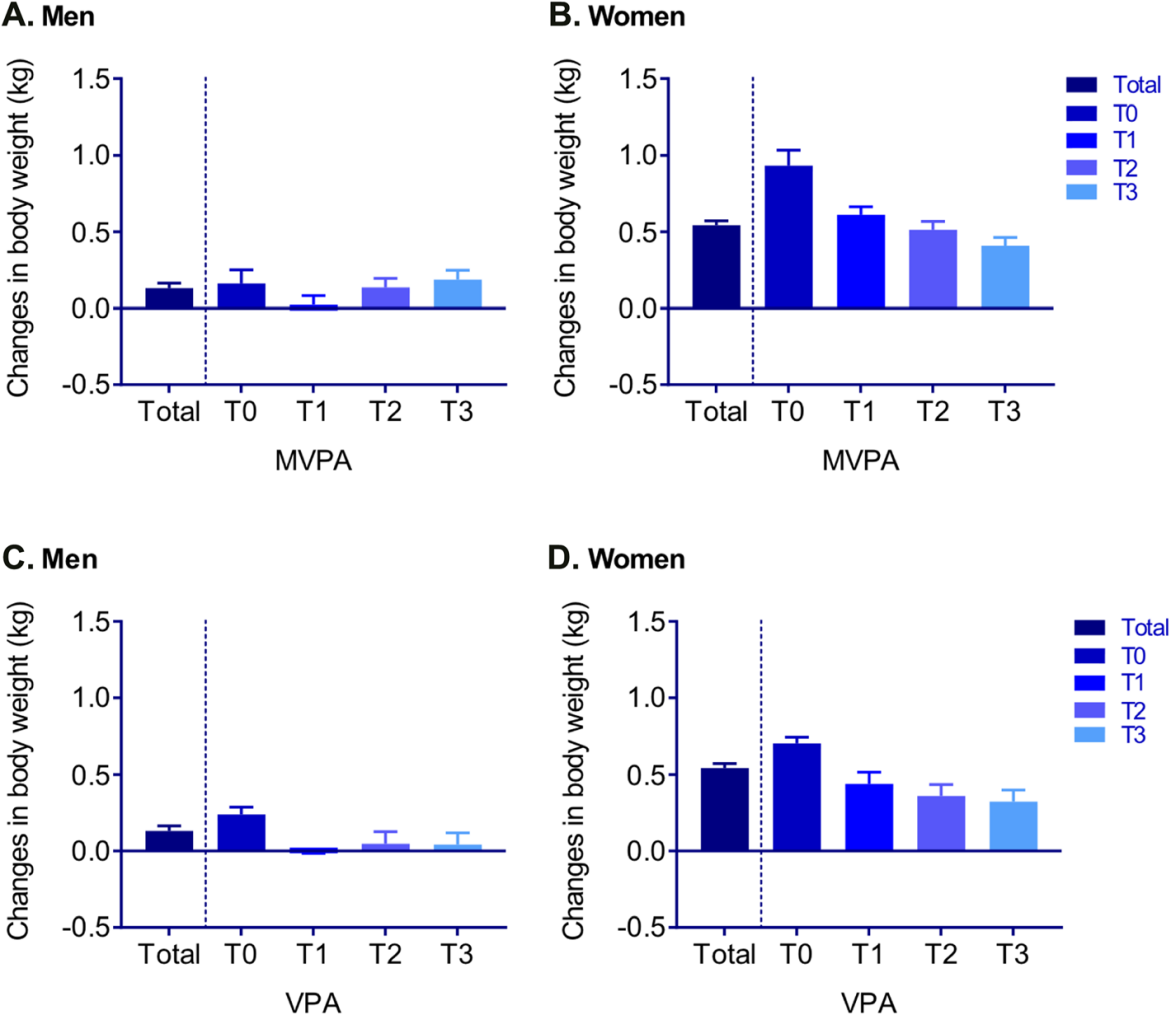
Estimated changes in body weight (kg) adjusted for age and education level, stratified by levels of moderate-to-vigorous (**a** and **b**) and vigorous (**c** and **d**) leisure-time physical activity. Measured body weight change adjusted with ANOVA. MVPA = moderate-to-vigorous physical activity, VPA = vigorous physical activity, T = tertile. Leisure-time MVPA and VPA were used in the analysis

After 4 years, the body weights of male and female participants had increased on average by 0.13 ± 0.03 kg and 0.45 ± 0.03 kg, respectively. Increases in the body weights of participants, stratified by age, were mostly observed in younger men (18–35 years) and young and middle-aged women (18–54 years, Figure S2). Changes in body weight, estimated with an age- and education-adjusted ANOVA were visualized according to PA levels in Fig. 2. All groups of the female participants gained body weight, but this increase in body weight was attenuated with increasing MVPA and VPA levels (T1–T3). This was observed in men only for the association between VPA and changes in body weight (Fig. 2).

To test the significance of these observations, inactive participants were compared to active participants in regression analyses (Table 2, Table S3). Higher leisure-time MVPA, as well as MPA and VPA separately, were associated with less gain in body weight in women. Beta coefficients (95%CI) for the MVPA> 0, MPA > 0 and VPA > 0 relative to each reference group (No-MVPA, No-MPA and No-VPA) were, respectively − 0.34 (− 0.56; − 0.13), − 0.32 (− 0.54; − 0.10) and − 0.30 (− 0.43; − 0.18) kg. These associations were dose-dependent when PA was categorized into four groups (Table 2, Table S3). In women, the beta-coefficients attenuated by 10–20% but remained significant after adjusting for potential confounders, including muscle mass. An in-depth investigation of the roles of the confounders indicated that the diet-based confounding effect was slightly stronger than the confounding effects of smoking and alcohol consumption (Table S2). In men, higher VPA, but not higher MPA or MVPA, was associated with less body weight gain. However, after adjusting for potential confounders, the association was no longer significant (Tables 2, S2, S3).

**Table 2:** Leisure-time physical activity and 4-year changes in body weight

Physical activity	Unstandardized beta coefficients kg body weight							
	Basic model		Model 1		Model 2		Model 3	
	B (95%CI)	*P*-value	B (95%CI)	*P*-value	B (95%CI)	*P*-value	B (95%CI)	*P*-value
**Men**								
MVPA-T0	0 (Reference)	–	0 (Reference)	–	0 (Reference)	–	0 (Reference)	–
MVPA-T1	− 0.14 (− 0.35; 0.07)	0.19	− 0.05 (− 0.26; 0.16)	0.61	− 0.03 (− 0.25; 0.19)	0.78	− 0.03 (− 0.24; 0.18)	0.80
MVPA-T2	− 0.03(− 0.24; 0.18)	0.81	0.10 (− 0.11; 0.31)	0.35	0.12 (− 0.10; 0.33)	0.30	0.13 (− 0.08; 0.34)	0.22
MVPA-T3	0.02 (− 0.19; 0.24)	0.82	0.19 (− 0.03; 0.40)	0.09	0.20 (−0.02; 0.42)	0.08	0.21 (−0.01; 0.43)	0.053
VPA-T0	0 (Reference)	–	0 (Reference)	–	0 (Reference)	–	0 (Reference)	–
VPA-T1	−0.25 (−0.42; − 0.09)	**0.03**	− 0.20 (− 0.37;-0.03)	0.02	−0.20 (− 0.37;-0.02)	0.03	−0.18 (− 0.35; − 0.01)	0.04
VPA-T2	−0.19 (− 0.38; − 0.01)	**0.04**	−0.11 (− 0.30; 0.07)	0.24	−0.09 (− 0.28; 0.11)	0.39	−0.09 (− 0.27; 0.10)	0.35
VPA-T3	−0.20 (− 0.38; − 0.02)	**0.03**	−0.09 (− 0.27; 0.09)	0.34	−0.10 (− 0.28; 0.09)	0.32	−0.07 (− 0.24; 0.11)	0.42
**Women**								
MVPA-T0	0 (Reference)	–	0 (Reference)	–	0 (Reference)	–	0 (Reference)	–
MVPA-T1	−0.32 (−0.55; − 0.10)	**0.005**	− 0.24 (− 0.47; − 0.02)	**0.036**	−0.25 (− 0.48; − 0.02)	**0.037**	−0.23 (− 0.45; − 0.02)	**0.048**
MVPA-T2	−0.42 (− 0.65; − 0.20)	**0.000**	−0.31 (− 0.53; − 0.08)	**0.008**	−0.35 (− 0.59; − 0.12)	**0.003**	−0.29 (− 0.52; − 0.07)	**0.012**
MVPA-T3	−0.53 (− 0.75; − 0.30)	**0.000**	−0.38 (− 0.61; − 0.16)	**0.001**	−0.42 (− 0.65; − 0.18)	**0.001**	−0.38 (− 0.60; − 0.14)	**0.001**
VPA-T0	0 (Reference)	–	0 (Reference)	–	0 (Reference)	–	0 (Reference)	–
VPA-T1	−0.27 (− 0.44; − 0.10)	**0.002**	−0.22 (− 0.39; − 0.05)	**0.011**	−0.24 (− 0.42; − 0.07)	**0.007**	−0.21 (− 0.38; − 0.04)	**0.015**
VPA-T2	−0.35 (− 0.51; − 0.18)	**0.000**	−0.30 (− 0.46; − 0.13)	**0.001**	−0.33 (− 0.51; − 0.16)	**0.000**	−0.29 (− 0.46; − 0.12)	**0.001**
VPA-T3	−0.38 (− 0.55; − 0.21)	**0.000**	−0.32 (− 0.49; − 0.15)	**0.000**	−0.33 (− 0.51; − 0.15)	**0.000**	−0.31 (− 0.49; − 0.14)	**0.000**

Regression analysis. Data on MPA is shown in Supplementary material, Table S3. Determinants are dummy exposure variables for physical activities for comparison between the reference group (No-MVPA, and No-VPA, T0) and tertiles of MVPA and VPA (T1–3). Data are expressed as unstandardized beta coefficient with 95% confidence interval (95% CI). MVPA = moderate-to-vigorous physical activity, VPA = vigorous physical activity, MPA = moderate physical activity. T = tertile

Basic model = age and education

Model 1 = Basic model + diet, smoking and alcohol use

Model 2 = Model 1 + 24-h urinary creatinine excretion

Model 3 = Model 1 + occupational MVPA

**Fig. 3:**
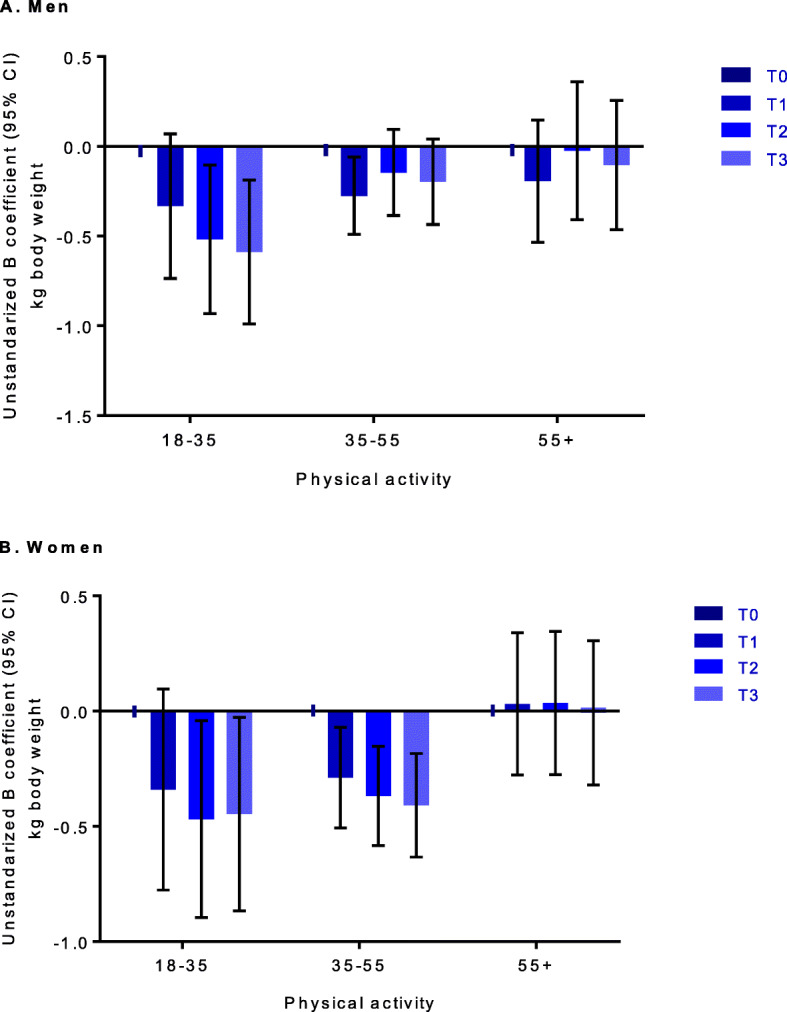
Association between vigorous leisure-time physical activity and changes in body weight, stratified by age in men (**a**) and women (**b**). Regression analysis. Data are expressed as unstandardized beta coefficient (presented as bar) with 95% confidence interval (95% CI, presented as arrow). Physical activity was shown as vigorous physical activity categories (T0-T3). T = tertile. Analysis was adjusted for age, education. Diet score, energy intake, smoking and alcohol use

Stratification of the participants by age revealed significant associations mainly in younger adults (Fig. 3). For men below 35 years and for women aged 35–55 years, leisure-time VPA was dose-dependently associated with less gain in body weight after fully adjusting for confounding factors (Table S4).

We conducted additional analyses aimed at elucidating the role of individual daily-life activities within the physical activity domain for translational purposes (Table S5). These analyses were performed for men below 35 years and women below 55 of age years because significant associations of LTPA and changes in body weight were observed for individuals in these age groups. Our findings based on analyses with dichotomized PA (No-MPA and MPA > 0 or No-VPA and VPA > 0) indicated that higher levels of moderate (cycling) and vigorous (cycling and sports) PA were associated with less weight gain in women after fully adjusting for confounding factors. For men, only higher levels of VPA (cycling and sports) were associated with less weight gain (Table S5). In a sensitivity analysis, there was no clear association between occupational MVPA and changes in body weight among both men and women (Table 2).

## Discussion

In this large-scale, population-based study, a higher leisure-time MVPA was found to be associated to less weight gain in women in a dose-dependent way. Moreover, these associations were stronger and independent of other potential confounders in women under the age of 55 years. Furthermore, the potentially favourable effects of PA for women applied to both moderate and vigorous physical activities like cycling and sports. Among male participants, strenuous physical activities, such as vigorous cycling and sports, were predominantly associated to lower weight gain but only in younger (< 35 years) men after adjusting for other lifestyle factors.

Several previous prospective studies found an inverse association between PA and changes in body weight [11–13, 32, 33]. However this association has not been confirmed in other studies [14–17]. Moreover, this association was found to be restricted to specific groups in some studies [18, 19]. For instance, a large-scale, multi-country EPIC study (*n* = 288,498) found an association between PA and 5-year changes in body weight only in younger women (< 50 years) and those of normal weight [18]. In our large-scale, population-based study, the benefits of LTPA differed among men and women relative to the PA intensity level. Moreover, the associations between PA and changes in body weight differed according age. These core findings are discussed in more detail below.

Clinical guidelines on PA levels state that physical activities at the moderate-to-vigorous level, but not at the light level, are essential for maintaining a healthy body weight [34]. However, most previous studies focused on total PA, including light PA, and did not test for or report on different PA intensity levels [14, 15, 18, 19, 21]. The few studies that tested intensity levels suggest that physical activities at higher intensity levels are more effective than those at lower intensity levels in weight management [17, 35]. Williams et al. found that weight loss from running exceeded that from walking after 6.2 years of follow-up [35]. Furthermore, there is some debate about intensity, duration and load. In theory, a longer bout of MPA would lead to the same energy expenditure as a shorter bout of VPA. However, a review study concluded that even if the PA requires the same amount of energy, so same energy expenditure but at moderate or vigorous load, there were different effects, suggesting a greater cardioprotective benefit from VPA than MPA [36]. More recent studies confirm this [37, 38]. In this observational study, we cannot provide explanatory mechanisms related to exact load or duration. Instead, we study the effects of MPA and VPA in free-living situations. The interpretation is that people who practice more VPA are less prone to weight gain. The value is in quantifying this association, describing relevant gender differences, and attributing this relation to the type of activity in a large population of participants.

A question that we aimed to address in our study was whether a vigorous level of PA better predicts future changes in body weight. This was found to be the case in men (< 35 years) for whom only vigorous activities were related to weight changes. However, in women, both moderate (cycling) and vigorous (cycling and sports) activities were related to body weight changes. Even more so, the benefits of total daily-life MVPA were mostly explained by MPA in women. The considerably lower changes in the body weights of men compared with those of women during the follow-up assessment may be indicative of a statistical power issue that could partly account for this gender difference. Another explanation could be that male participants’ reporting of VPA was more accurate than their reporting of MPA or other physical activities in the questionnaire [39]. Accordingly, more longitudinal studies are needed to establish the effects of different PA intensities for men to prevent body weight gain. For women, not only VPA but also MPA can be considered as an option for avoiding body weight gain.

In this study, the association of physical activity with changes in body weight was mainly observed in younger adults. This association may be related to the observation that life-time weight gain mostly occurred during this period (Figure S2). In line with our findings, another study found that the transition from normal weight to obesity was mostly observed around the ages of 28–33 and 31–36 years [33]. The findings of this and other studies suggest that a high level of activity during those ages can prevent overweight or obesity [11, 33]. This is plausible, as an increased energy expenditure may help to avoid a positive energy balance. Nevertheless, the usefulness of physical activity alone in body weight control has been doubted. This is based on studies showing that increasing physical activity alone is not effective for weight loss [10]. In our view, the process of weight loss is not simply the reverse of the process of weight gain, as with weight loss, there are many hormones counteracting negative energy balance [40]. In the case of prevention of weight gain, these hormones do not play a role. Indeed, a number of studies have reported that a very active lifestyle at younger adult ages may entail the benefit of obesity prevention at later ages [9, 12, 30]. Moreover, a higher BMI in early adult life is a predictor of cardiovascular diseases in later life [41]. Thus, a conclusion that merits emphasis is that increasing PA at younger ages may be an important primordial obesity prevention strategy while simultaneously preventing non-communicable diseases in later adult life.

It should be noted that our outcome measure focused on changes in overall body weight and not specifically on body fat mass. Changes in body weight, especially in younger adults, could reflect changes in muscle mass. Consequently, we adjusted for creatinine excretion in all of the analyses and found that the association between PA and changes in body weight was independent of changes in muscle mass over time. Previous studies that used direct measures of body composition indeed explained differences in weight gain by smaller changes in body fat in very active younger adults and greater gains in body fat in inactive young adults [11, 42]. Although 24-h urinary creatinine excretion may not be a precise marker for the absolute level of muscle mass, changes in creatinine excretion have been found to be a more sensitive measure for changes in body composition compared with DEXA [43]. Therefore, we concluded that increased PA can be an effective strategy for preventing body weight gain independently of muscle mass.

The main strength of our study is its large sample size obtained from the general population, which enabled us to estimate the dose-dependency of different PA intensities with changes in body weight for sex- and age-differentiated groups with sufficient statistical power. A second strength of the study relates to the objective measurements of body weight that were taken during the baseline and follow-up phases. Although we excluded many participants who are obese or with several diseases it helps to minimize cause-effect bias relating to changes in PA or body weight caused by poor health. However, it also can be a limitation in that it confers a reduction of representativeness of the population. Another limitation is that PA was reported only at the baseline stage. A few studies have concluded that a single measure of PA weakly predicts future changes in body weight [14, 44], which may be related to a bidirectional association of PA and obesity [17, 45]. The inclusion in the analyses of more individuals living with obesity could attenuate the association between baseline PA and body weight at the follow-up assessment because individuals with obesity may on the one hand may be more inclined to be y inactive while simultaneously making conscious efforts to prevent weight gain through diet. In our study, we included only non-obese individuals with the aim of reducing such information bias. Another limitation relates to our assessment of PA that was based on self-reporting and therefore subject to recall bias. However, the SQUASH questionnaire has been validated within the general population, demonstrating a Spearman correlation coefficient for reproducibility of 0.58 [25]. Furthermore, the SQUASH questionnaire was not specifically validated for the different types of leisure time activity. Although PA quantification may have been subject to reporting bias, the qualitative information about the types and domain of MVPA proved valuable.

## Conclusions

A higher level of leisure-time MVPA is associated with less gain in body weight in women. The potentially favourable effects of MVPA for women applied to both moderate and vigorous physical activities. The associations were found to be dose-dependent, suggesting that more MVPA is more beneficial. Furthermore, the associations were strongest in younger and middle-aged women (< 55 years) and were independent of diet, smoking, alcohol use, and 4-year changes in creatinine excretion, considered a marker of muscle mass. For men, only vigorous LTPA was associated with less weight gain in younger adults (< 35 years).

## Supplementary Information

**Additional file 1.** STROBE Statement—checklist of items that should be included in reports of observational studies.

**Additional file 2: **Anthropometry and laboratory measurements. Definition of lifestyle confounders and diseases. **Table S1.** General characteristics of the study population, by age. **Table S2.** Role of lifestyle confounders in the association between physical activity and changes in body weight. **Table S3.** Leisure-time MPA and 4-year changes in body weight. **Table S4.** Moderate-to-vigorous physical activity and changes in body weight, according to age. **Table S5.** Individual physical activities and 4-year changes in body weight. **Fig. S1.** Level of daily-life physical activity according to sex. **Fig. S2.** 4-year changes in body weight, according to 6 categories of age.

## Data Availability

The Lifelines Cohort does not enable public data sharing. The cohort’s data is only available to researchers who, upon approval of a submitted research proposal, have signed a Data/Material Transfer Agreement.
