# Supplementary material for: Physical activity and 4-year changes in body weight in 52,498 non-obese people: the Lifelines cohort
Source: Int J Behav Nutr Phys Act. 2021 Jun 7;18:75. doi: 10.1186/s12966-021-01141-8 (PMC8186174; doi:10.1186/s12966-021-01141-8)
Supplement: Supplementary file 2 — Additional file 2: Anthropometry and laboratory measurements. Definition of lifestyle confounders and diseases. Table S1. General characteristics of the study population, by age. Table S2. Role of lifestyle confounders in the association between physical activity and changes in body weight. Table S3. Leisure-time MPA and 4-year changes in body weight. Table S4. Moderate-to-vigorous physical activity and changes in body weight, according to age. Table S5. Individual physical activities and 4-year changes in body weight. Fig. S1. Level of daily-life physical activity according to sex. Fig. S2. 4-year changes in body weight, according to 6 categories of age. [file 12966_2021_1141_MOESM2_ESM.docx]

**SUPPLEMENTARY MATERIAL**

**Title: Physical activity and 4-year changes in body weight in 52,498 non-obese people: The Lifelines Cohort**

Oyuntugs Byambasukh,^1,3^ Petra Vinke^1^, Daan Kromhout^1^, Gerjan Navis^2^, Eva Corpeleijn^1^

^1^Department of Epidemiology, University Medical Center Groningen, University of Groningen, Groningen, the Netherlands, ^2^Department of Internal Medicine, University Medical Center Groningen, University of Groningen, Groningen, the Netherlands, ^3^Department of Internal Medicine, Mongolian National University of Medical Sciences, Ulaanbaatar, Mongolia

**Correspondence details:**

Oyuntugs Byambasukh

Unit of Lifestyle Medicine in Obesity and Diabetes

Department of Epidemiology (FA40)

University Medical Center Groningen

University of Groningen

P.O. Box 30 001, 9700 RB Groningen, The Netherlands

Tel: +31(0)641152211, E-Mail: [o.byambasukh@umcg.nl](mailto:o.byambasukh@umcg.nl), [oyuntugs@mnums.edu.mn](mailto:oyuntugs@mnums.edu.mn)

**Contents:**

| **Supplementary method:** | | **Page** |
| --- | --- | --- |
| Method 1. | Anthropometry and laboratory tests | 1 |
| Method 2. | Definitions of covariates | 1-2 |

| **Supplementary tables:** | | **Page** |
| --- | --- | --- |
| Table S1. | General characteristics of the study population, by age | 3 |
| Table S2. | Role of lifestyle related confounders in the association between physical activity and changes in body weight | 4 |
| Table S3 | Leisure-time MPA and 4-year changes in body weight | 4 |
| Table S4 | Moderate-to-vigorous physical activity and changes in body weight, according to age | 5 |
| Table S5 | Individual physical activities and 4-year changes in body weight | 5 |

| **Supplementary figures:** | | **Page** |
| --- | --- | --- |
| Figure S1. | Level of daily-life physical activity according to sex | 6 |
| Figure S2. | 4-year changes in body weight, according to 6 categories of age | 7 |

**1. Anthropometry and laboratory measurements**

Body height, waist circumference, and blood pressure were measured by a permanent staff of well-trained assistants using a standardized protocol. Height was measured with a stadiometer placing the heels against the rod and the head in Frankfort Plane position. Waist circumference was measured in standing position with a tape measure all around the body, at the level midway between the lower rib margin and the iliac crest. Body mass index (BMI) was calculated as weight (kg) divided by height squared (m^2^).[1][2]

The blood samples were collected in the fasting state, between 8.00 and 10.00 a.m. and analyzed on the day of collection at the Department of Laboratory Medicine of the University Medical Center Groningen, the Netherlands. Participants were requested to fast for at least 12 hours prior to the blood draw. Fasting plasma glucose (FPG) was measured by the hexokinase method. Serum levels of total and HDL cholesterol were measured using an enzymatic colorimetric method, triglycerides using a colorimetric UV method on a Roche Modular P chemistry analyzer (Roche, Basel, Switzerland).[1][2]

**2. Definition of lifestyle confounders and diseases**

Education level: Education was categorized as low (no education, primary education, lower or preparatory vocational education and lower general secondary education), medium (intermediate vocational education or apprenticeship, higher general senior secondary education or pre-university secondary education) and high (higher vocational education and university).

Current smoking: Smoking status was categorized as non-smokers and smokers. Non-smokers were those who had not smoked during the last month and had also never smoked for longer than a year.

Daily caloric intake and alcohol intake: From the Food Frequency Questionnaire, daily caloric intake and alcohol intake were calculated and presented as kilocalories a day (kcal/day) and grams of alcohol a day (gr/day). The calculation was based on intake frequency and the average number of units consumed on a day (divided the number of alcoholic drinks/week by 7). In the Netherlands a standard unit contains 9.9 grams of alcohol. For each type of alcoholic beverage, respondents indicated whether they consumed it never (0%), sometimes (30%), often (70%) or always (100%).[1][2]

The Lifelines Diet Score: Based on food groups derived from the food frequency questionnaire, diet quality was scored by The Lifelines Diet Score (LLDS). The LLDS is based on the international scientific evidence on diet and chronic disease relations from prospective cohort analyses and randomized controlled trials, summarized by the Dutch Health Council and underlying the 2015 Dutch dietary guidelines. The LLDS ranks the relative intake of nine food groups for which there is strong international peer-reviewed scientific evidence of positive health effects (vegetables, fruit, whole grain products, legumes & nuts, fish, oils & soft margarines, unsweetened dairy, coffee and tea) and three food groups for which there is strong international peer-reviewed scientific evidence of negative health effects (red & processed meat, butter & hard margarines and sugar-sweetened beverages). For each of the food groups, quintiles of consumption in grams/1000 kcal are determined and awarded zero to four points. For the positive food groups, higher scores are awarded to higher quintiles of consumption, whereas intake for negative food groups is scored inversely. The sum of these components leads to a LLDS between zero and 48.[3]

References:

1. Stolk RP, Rosmalen JGM, Postma DS, de Boer RA, Navis G, Slaets JPJ, et al. Universal risk factors for multifactorial diseases: LifeLines: a three-generation population-based study. Eur J Epidemiol. 2008;23: 67±74. doi: 10.1007/s10654-007-9204-4 PMID: 18075776.
2. Scholtens S, Smidt N, Swertz MA, Bakker SJL, Dotinga A, Vonk JM, et al. Cohort Profile: LifeLines, a three-generation cohort study and biobank. Int J Epidemiol. 2015; 44: 1172±1180. doi: 10.1093/ije/dyu229 PMID: 25502107.
3. Vinke PC, Corpeleijn E, Dekker LH, Jacobs DR, Navis G, Kromhout D: Development of the food-based Lifelines Diet Score (LLDS) and its application in 129,369 Lifelines participants. Eur J Clin Nutr 2018;72:1111–1119.

**Table S1.** General characteristics of the study population, by age

| **Characteristics** | **18-34** | **35-54** | **≥55+** | **P-value** |
| --- | --- | --- | --- | --- |
| **Men** |  |  |  |  |
| Number (%) | 5,530 (24.2) | 13,027 (57.1) | 4,270 (18.7) | - |
| Age (years) | 29 (26-32) | 45 (41-49) | 62 (59-66) | - |
| Education: Low, (%, n) | 13.9 (769) | 24.7 (3224) | 38.3 (1637) | <0.001 |
| Current smoking, (%, n) | 27.9 (1544) | 20.0 (2605) | 12.8 (546) | <0.001 |
| Alcohol use, (gr/day) | 7.6 (3.2-15.8) | 6.7 (2.6-13.9) | 8.2 (3.0-17.0) | <0.001 |
| Lifelines Diet score | 20.8 ± 5.4 | 22.8 ± 5.4 | 25.2 ± 5.5 | <0.001 |
| Energy intake (kcal/day) | 2507.3 ± 653.4 | 2440.6 ± 614.8 | 2173.0 ± 534.5 | <0.001 |
| Body weight (kg) | 82.5 ± 10.6 | 86.1 ± 9.8 | 83.4 ± 9.0 | <0.001 |
| BMI (kg/m^2^) | 24.2 ± 2.6 | 25.5 ± 2.4 | 25.7 ± 2.2 | <0.001 |
| Waist circumference (cm) | 87.6 ± 8.2 | 93.1 ± 7.6 | 95.2 ± 7.4 | <0.001 |
| Total cholesterol (mmol/L) | 4.6 ± 0.9 | 5.3 ± 0.9 | 5.5 ± 0.9 | <0.001 |
| HDL-cholesterol | 1.3 ± 0.3 | 1.3 ± 0.3 | 1.4 ± 0.3 | <0.001 |
| Triglycerides (mmol/L) | 1.0 (0.7-1.4) | 1.2 (0.8-1.6 | 1.1 (0.8-1.5) | <0.001 |
| Plasma glucose (mmol/L) | 4.9 ± 0.4 | 5.0 ± 0.5 | 5.2 ± 0.5 | <0.001 |
| Leisure-time MVPA |  |  |  |  |
| No MVPA, (%, n) | 12.2 (676) | 14.6 (1902) | **10.7 (457)** | **<0.001** |
| MVPA (min/week) | 225.5 (80-420) | 180 (60-360) | **270 (120-495)** | **<0.001** |
| Leisure-time VPA |  |  |  |  |
| No VPA, (%, n) | 35.7 (1976) | 48.1 (6263) | **52.6 (2245)** | **<0.001** |
| VPA (min/week) | 90 (0-270) | 40 (0-180) | **0 (0-180)** | **<0.001** |
| Occupational MVPA (min/week) | 270.1 (60-520) | 220.1 (72-380) | 76 (60-395) | <0.001 |
| **Women** |  |  |  |  |
| Number (%) | 6,046 (20.4) | 18,201 (61.3) | 5,424 (18.3) | - |
| Age (years) | 27 (22-32) | 45 (41-49) | 61 (58-65) | - |
| Education: Low, (%, n) | 11.8 (713) | 23.1 (4199) | 55.5 (3012) | <0.001 |
| Current smoking, (%, n) | 23.4 (1412) | 17.9 (3250) | 10 (544) | <0.001 |
| Alcohol use, (gr/day) | 2.6 (0.7-6.4) | 2.8 (0.7-7.1) | 3.6 (0.8-9.9) | <0.001 |
| Lifelines Diet score | 22.7 ± 5.9 | 25.4 ± 5.8 | 28.2 ± 5.5 | <0.001 |
| Energy intake (kcal/day) | 1859.5 ± 464.6 | 1890.7 ± 460.5 | 1766.3 ± 417.4 | <0.001 |
| Body weight (kg) | 68.2 ± 9.4 | 70.4 ± 8.9 | 69.5 ± 8.3 | <0.001 |
| BMI (kg/m^2^) | 23.3 ± 2.8 | 24.3 ± 2.7 | 25.0 ± 2.6 | <0.001 |
| Waist circumference (cm) | 79.3 ± 8.7 | 83.3 ± 8.5 | 86.4 ± 8.5 | <0.001 |
| Total cholesterol (mmol/L) | 4.4 ± 0.8 | 5.1 ± 0.9 | 5.9 ± 1.0 | <0.001 |
| HDL-cholesterol | 1.6 ± 0.3 | 1.7 ±0.4 | 1.8 ± 0.4 | <0.001 |
| Triglycerides (mmol/L) | 0.8 (0.6-1.1) | 0.8 (0.6-1.1) | 1.0 (0.8-1.3) | <0.001 |
| Plasma glucose (mmol/L) | 4.6 ± 0.4 | 4.8 ± 0.4 | 4.9 ± 0.5 | <0.001 |
| Leisure-time MVPA |  |  |  |  |
| No MVPA, (%, n) | 7.4 (447) | 9.6 (1748) | 7.4 (402) | <0.001 |
| MVPA (min/week) | 210 (90-380) | 190(80-360) | 270 (120-480) | <0.001 |
| Leisure-time VPA |  |  |  |  |
| No VPA, (%, n) | 44.8 (2710) | 57.2 (10416) | 47.9 (2597) | <0.001 |
| VPA (min/week) | 45 (0-150) | 0 (0-100) | 30 (0-120) | <0.001 |
| Occupational MVPA (min/week) | 120 (40-320) | 115.3 (60-280) | 36 (70-285) | <0.001 |

Data are presented as mean ± SD or median (25th to 75th percentile) and number (percentages, %). Abbreviations: BMI=body mass index, BP=blood pressure, HDL-C=high-density lipoprotein cholesterol, HbA1c=hemoglobin-A1c, MVPA=moderate-to-vigorous physical activity, VPA=vigorous physical activity, T=tertile.

**Table S2.** Role of lifestyle confounders in the association between physical activity and changes in body weight

| **PA** | **Unstandardized beta coefficients kg body weight** | | | | | |
| --- | --- | --- | --- | --- | --- | --- |
|  | **Basic model** | | **Model 1A** |  | **Model 1B** |  |
|  | B (95%CI) | *P* | B (95%CI) | *P* | B (95%CI) | *P* |
| **Men** | | | | | | |
| Moderate-to-vigorous PA | | | | | | |
| T0 | 0 (Reference) | - | 0 (Reference) | - | 0 (Reference) | - |
| T1 | -0.140 (-0.349; 0.069) | 0.19 | -0.097 (-0.306; 0.112) | 0.37 | -0.092 (-0.301; 0.117) | 0.39 |
| T2 | -0.026(-0.235; 0.183) | 0.81 | 0.043 (-0.167; 0.252) | 0.69 | 0.038 (-0.171; 0.248) | 0.72 |
| T3 | 0.024 (-0.187; 0.235) | 0.82 | 0.120 (-0.092; 0.333) | 0.27 | 0.099 (-0.113; 0.311) | 0.36 |
| Vigorous PA | | | | | | |
| T0 | 0 (Reference) | - | 0 (Reference) | - | 0 (Reference) | - |
| T1 | -0.254 (-0.423; -0.085) | **0.03** | -0.223 (-0.392;-0.054) | **0.01** | -0.228 (-0.397;-0.059) | **0.008** |
| T2 | -0.192 (-0.376; -0.008) | **0.04** | -0.144 (-0.329; 0.040) | 0.13 | -0.154 (-0.338; 0.030) | 0.10 |
| T3 | -0.197 (-0.377; -0.018) | **0.03** | -0.131 (-0.312; 0.049) | 0.15 | -0.147 (-0.327; 0.032) | 0.11 |
| **Women** | | | | | | |
| Moderate-to-vigorous PA | | | | | | |
| T0 | 0 (Reference) | - | 0 (Reference) | - | 0 (Reference) | - |
| T1 | -0.323 (-0.547; -0.099) | **0.005** | -0.260 (-0.485; -0.035) | **0.023** | -0.298 (-0.523; -0.073) | **0.009** |
| T2 | -0.420 (-0.645; -0.195) | **0.000** | -0.339 (-0.565; -0.113) | **0.003** | -0.380 (-0.606; -0.154) | **0.001** |
| T3 | -0.525 (-0.750; -0.300) | **0.000** | -0.425 (-0.652; -0.198) | **0.000** | -0.475 (-0.701; -0.249) | **0.000** |
| Vigorous PA | | | | | | |
| T0 | 0 (Reference) | - | 0 (Reference) | - | 0 (Reference) | - |
| T1 | -0.266 (-0.435; -0.097) | **0.002** | -0.243 (-0.412; -0.074) | **0.005** | -0.238 (-0.407; -0.068) | **0.006** |
| T2 | -0.345 (-0.512; -0.178) | **0.000** | -0.321 (-0.489; -0.154) | **0.000** | -0.315 (-0.483; -0.148) | **0.000** |
| T3 | -0.382 (-0.552; -0.212) | **0.000** | -0.346 (-0.517; -0.175) | **0.000** | -0.348 (-0.518; -0.177) | **0.000** |

Regression analysis. Determinants are dummy exposure variables for physical activities for comparison between the reference group (No-MVPA, and No-VPA, T0) and tertiles of MVPA and VPA (T1-3). Data are expressed as unstandardized beta coefficient with 95% confidence interval (95% CI). PA=physical activity, T=tertile.

Basic model=age and education.

Model 1A = Basic model + diet score.

Model 1B = Basic model + smoking and alcohol use.

**Table S3.** Leisure-time MPA and 4-year changes in body weight

| Physical activity | | **Unstandardized beta coefficients kg body weight** | | | | | |
| --- | --- | --- | --- | --- | --- | --- | --- |
|  |  | **Basic model** | | **Model 1** |  | **Model 2** |  |
|  |  | B (95%CI) | *P*-value | B (95%CI) | *P*-value | B (95%CI) | *P*-value |
| **Men** | | | | | | | |
|  | MPA-T0 | 0 (Reference) | - | 0 (Reference) | - | 0 (Reference) | - |
|  | MPA-T1 | -0.26 (-0.48; -0.03) | **0.02** | -0.05 (-0.37; 0.07) | 0.19 | -0.11 (-0.33; 0.11) | 0.32 |
|  | MPA-T2 | -0.02 (-0.25; 0.21) | 0.85 | 0.10 (-0.13; 0.34) | 0.38 | 0.13 (-0.10; 0.36) | 0.27 |
|  | MPA-T3 | 0.18 (-0.05; 0.41) | 0.13 | 0.32 (0.09; 0.55) | **0.007** | 0.34 (0.11; 0.56) | **0.004** |
| **Women** | | | | | | | |
|  | MPA-T0 | 0 (Reference) | - | 0 (Reference) | - | 0 (Reference) | - |
|  | MPA-T1 | -0.39 (-0.62; -0.15) | **0.001** | -0.29 (-0.53; -0.05) | **0.018** | -0.28 (-0.52; -0.04) | **0.022** |
|  | MPA-T2 | -0.43 (-0.67; -0.20) | **0.000** | -0.31 (-0.55; -0.07) | **0.011** | -0.30 (-0.54; -0.06) | **0.014** |
|  | MPA-T3 | -0.50 (-0.73; -0.27) | **0.000** | -0.36 (-0.60; -0.13) | **0.003** | -0.36 (-0.59; -0.12) | **0.003** |

Regression analysis. Determinants are dummy exposure variables for physical activities for comparison between the reference group (No-MPA, T0) and tertiles of MPA (T1-3). Data are expressed as unstandardized beta coefficient with 95% confidence interval (95% CI). MPA=moderate physical activity, T=tertile.

Basic model=age and education.

Model 1 = Basic model + diet, smoking and alcohol use.

Model 2 = Model 1 + 24-hour urinary creatinine excretion

**Table S4.** Moderate-to-vigorous physical activity and changes in body weight, according to age

| **PA** | **Unstandardized beta coefficients kg body weight** | | | | | |
| --- | --- | --- | --- | --- | --- | --- |
|  | **18-34 years** |  | **35-54 years** |  | **≥55 years** |  |
|  | B (95%CI) | P-value | B (95%CI) | P-value | B (95%CI) | P-value |
| **Men** |  |  |  |  |  |  |
| MVPA-T0 | 0 (Reference) | - | 0 (Reference) | - | 0 (Reference) | - |
| MVPA-T1 | -0.255 (-0.477; 0.033) | 0.02 | -0.151(-0.374; 0.072) | 0.19 | -0.113 (-0.334; 0.109) | 0.32 |
| MVPA-T2 | -0.024 (-0.253; 0.208) | 0.85 | 0.104 (-0.128; 0.336) | 0.38 | -0.130 (-0.100; 0.360) | 0.27 |
| MVPA-T3 | 0.177 (-0.051; 0.405) | 0.13 | 0.316 (0.086; 0.546) | 0.01 | 0.337 (0.109; 0.565) | 0.01 |
| VPA-T0 | 0 (Reference) | - | 0 (Reference) | - | 0 (Reference) | - |
| VPA-T1 | -0.242 (-0.645; 0.161) | 0.24 | -0.214 (-0.430; 0.001) | 0.051 | -0.134 (-0.475; 0.208) | 0.44 |
| VPA-T2 | -0.395 (-0.810; 0.020) | 0.06 | -0.056 (-0.297; 0.184) | 0.65 | 0.040 (-0.346; 0.426) | 0.84 |
| VPA-T3 | -0.473(-0.878; -0.068) | **0.02** | -0.074 (-0.314; 0.166) | 0.55 | -0.024 (-0.387; 0.339) | 0.90 |
| **Women** |  |  |  |  |  |  |
| MVPA-T0 | 0 (Reference) | - | 0 (Reference) | - | 0 (Reference) | - |
| MVPA-T1 | -0.386 (-0.624; -0.148) | **0.001** | -0.289 (-0.529; -0.049) | **0.018** | -0.278 (-0.517; -0.039) | **0.022** |
| MVPA-T2 | -0.433 (-0.670; -0.196) | **0.000** | -0.310 (-0.550; -0.071) | **0.011** | -0.298 (-0.535; -0.060) | **0.014** |
| MVPA-T3 | -0.500 (-0.733; -0.267) | **0.000** | -0.361 (-0.597; -0.125) | **0.003** | -0.355 (-0.590; -0.120) | **0.003** |
| VPA-T0 | 0 (Reference) | - | 0 (Reference) | - | 0 (Reference) | - |
| VPA-T1 | -0.313 (-0.750; 0.125) | 0.16 | -0.217 (-0.436; 0.002) | 0.052 | -0.074 (-0.236; 0.383) | 0.64 |
| VPA-T2 | -0.428 (-0.857; 0.002) | 0.051 | -0.296 (-0.512; -0.080) | **0.007** | -0.057 (-0.254; 0.368) | 0.72 |
| VPA-T3 | -0.383 (-0.807; 0.042) | 0.077 | -0.314 (-0.540; -0.088) | **0.006** | -0.015 (-0.299; 0.330) | 0.92 |
|  |  |  |  |  |  |  |

Regression analysis. Determinants are dummy exposure variables for physical activities for comparison between the reference group (No-MVPA and No-VPAT0) and tertiles of MVPA and VPA (T1-3). Data are expressed as unstandardized beta coefficient with 95% confidence interval (95% CI). MVPA=moderate-to-vigorous physical activity, VPA=vigorous physical activity, T=tertile.

Analysis was adjusted for age, education. diet score, smoking and alcohol use.

**Table S5.** Individual physical activities and 4-year changes in body weight

| **Leisure-time physical activity** | **Unstandardized beta coefficients kg body weight** **(95% CI)** | | | | | | |
| --- | --- | --- | --- | --- | --- | --- | --- |
|  | Basic model | | |  | Model 1 | | |
|  | PA=0  (Ref) | PA>0 | *P*-value |  | PA=0  (Ref) | PA>0 | *P*-value |
| **Men (<35 y)** |  |  |  |  |  |  |  |
| Walking (moderate) | 0 | -0.14 (-0.89; 0.21) | 0.71 |  | 0 | -0.04 (-0.81; 0.73) | 0.92 |
| Cycling at moderate | 0 | -0.01 (-0.48; 0.02) | 0.96 |  | 0 | 0.14 (-0.33; 0.61) | 0.56 |
| Cycling at vigorous | 0 | -0.74 (-1.15;-0.32) | **0.001** |  | 0 | -0.61 (-1.03;-0.19) | **0.001** |
| Sports at moderate | 0 | 0.12 (-0.46; 0.69) | 0.69 |  | 0 | 0.34 (-0.24; 0.92) | 0.25 |
| Sports at vigorous | 0 | -0.44 (-0.76;-0.12) | **0.01** |  | 0 | -0.35 (-0.67;-0.02) | **0.04** |
| Odd jobs (moderate) | 0 | 0.21 (-0.54; 0.95) | 0.59 |  | 0 | 0.29 (-0.46; 1.05) | 0.45 |
| **Women (<55 y)** |  |  |  |  |  |  |  |
| Walking (moderate) | 0 | -0.44 (-0.74;-0.13) | **0.01** |  | 0 | -0.23 (-0.54; 0.09) | 0.17 |
| Cycling at moderate | 0 | -0.42 (-0.65;-0.18) | **0.001** |  | 0 | -0.27 (-0.51;-0.03) | **0.03** |
| Cycling at vigorous | 0 | -0.45 (-0.66;-0.25) | **0.001** |  | 0 | -0.37 (-0.57;-0.16) | **0.001** |
| Sports at moderate | 0 | -0.39 (-0.66;-0.12) | **0.01** |  | 0 | -0.26 (-0.54; 0.01) | 0.058 |
| Sports at vigorous | 0 | -0.38 (-0.52;-0.24) | **0.001** |  | 0 | -0.32 (-0.46;-0.17) | **0.001** |
| Odd jobs (moderate) | 0 | -0.03 (-0.48; 0.42) | 0.89 |  | 0 | -0.23 (-0.24; 0.69) | 0.34 |

Regression analysis. Determinants are dummy exposure variables for physical activities for comparison between the reference group (No-MVPA or No-VPA) and MVPA>0 (VPA>0). Data are expressed as unstandardized beta coefficient or odds ratio with 95% confidence interval (95% CI). MVPA=moderate-to-vigorous physical activity. Analysis was adjusted for age, education. diet score, smoking and alcohol use.

Basic model=age and education.

Model 1 = Basic model + diet, smoking and alcohol use.


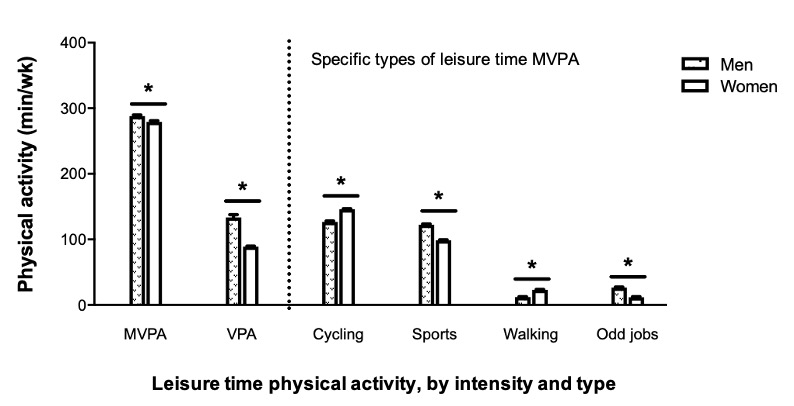


**Figure S1.** Level of daily-life physical activity according to sex.

MVPA (min/week) are expressed as adjusted means (adjusted for age, gender, education) for total and domain-specific physical activities. *indicates significant difference between men and women at *p* value < 0.05. MVPA=moderate-to-vigorous physical activity.

**Figure S2.** 4-year changes in body weight, according to 6 categories of age
